# Validation List no. 224. Valid publication of new names and new combinations effectively published outside the IJSEM

**DOI:** 10.1099/ijsem.0.006801

**Published:** 2025-07-31

**Authors:** Aharon Oren, Markus Göker

**Affiliations:** 1 The Institute of Life Sciences, The Hebrew University of Jerusalem, The Edmond J. Safra Campus, 9190401 Jerusalem, Israel; 2 Leibniz Institute DSMZ – German Collection of Microorganisms and Cell Cultures, Inhoffenstrasse 7B, 38124 Braunschweig, Germany

The purpose of this list is to publish in the International Journal of Systematic and Evolutionary Microbiology (IJSEM) those new names and new combinations that were effectively published outside the IJSEM as set forth in Rule 27 of the International Code of Nomenclature of Prokaryotes (ICNP) (2022 Revision) [1]. Authors and other individuals wishing to have new names and/or combinations included in future lists should send an electronic copy of the published paper to the IJSEM Editorial Office for confirmation that all the other requirements for valid publication have been met. It is also a requirement of IJSEM and the ICNP that authors of new species, new subspecies and new combinations provide evidence that types are deposited in two publicly accessible culture collections in two different countries. It should be noted that the date of valid publication of these new names and combinations is the date of publication of this list, not the date of the original publication of the names and combinations. The authors of the new names and combinations are as given below.

Inclusion of a name on these lists validates the publication of the name and, thus, makes it available in the nomenclature of prokaryotes. Inclusion of a name on these lists is part of the requirements of valid publication of an effectively published name. Indeed, some of these names may, in time, be considered later synonyms or it may be proposed to transfer the organisms to another genus, thereby necessitating the creation of a new combination. Conversely, it may be proposed to transfer an organism back to an earlier genus and to regard the basonym or an earlier new combination as the correct name instead of a more recent new combination. The ICNP does not decide on such taxonomic opinions.

**Table:** 

Name/author	Proposed as	Nomenclatural type^1^	Priority^2^	Reference
*Aeromonas ichthyocola* Ajmi *et al*. 2025, 8^3^	sp. nov.	A-5 (=DSM 117488=LMG 33534)	43	[2]
*Aeromonas mytilicola* Ajmi *et al*. 2025, 8^3^	sp. nov.	A-7 (=DSM 117490=LMG 33536)	43	[2]
*Aeromonas mytilicola* subsp. *aquatica* Ajmi *et al*. 2025, 8^3^	subsp. nov.	A-8 (=DSM 117493=LMG 33537)	43	[2]
*Aeromonas mytilicola* subsp. *mytilicola* Ajmi *et al*. 2025, 8^3,4^	subsp. nov.	A-7 (=DSM 117490=LMG 33536)	43	[2]
*Algirhabdus* Nedashkovskaya *et al*. 2025, 13^3^	gen. nov.	*Algirhabdus cladophorae*	33	[3]
*Algirhabdus cladophorae* Nedashkovskaya *et al*. 2025, 14^3^	sp. nov.	7Alg 153 (=KCTC 72606=KMM 6494)	33	[3]
*Anaerococcus cruorum* Boiten *et al*. 2025, 6^3^	sp. nov.	ENR1039 (=CCUG 77489=DSM 117235)	14	[4]
*Anaerococcus groningensis* Boiten *et al*. 2025, 6^3,5^	sp. nov.	ENR1011 (=CCUG 77488=DSM 117232)	14	[4]
*Anaerococcus kampingae* corrig. Boiten *et al*. 2025, 6^3,6^	sp. nov.	ENR0874 (=CCUG 77487=DSM 117234)	14	[4]
*Anaerococcus martiniensis* Boiten *et al*. 2025, 6^3,7^	sp. nov.	ENR0831 (=CCUG 77486=DSM 117233)	14	[4]
*Aquirufa avitistagni* Pitt *et al*. 2025, 10^3^	sp. nov.	OSTEICH-129V (=DSM 118088=JCM 37100)	20	[5]
*Aquirufa echingensis* Pitt *et al*. 2025, 10^3^	sp. nov.	PLAD-142S6K (=DSM 117799=JCM 37096)	20	[5]
*Aquirufa esocilacus* Pitt *et al*. 2025, 9^3^	sp. nov.	HETE-83D (=DSM 118087=JCM 37094)	20	[5]
*Aquirufa originis* Pitt *et al*. 2025, 9^3^	sp. nov.	KTFRIE-69F (=DSM 117798=JCM 37095)	20	[5]
*Blastococcus goldschmidtiae* Nouioui *et al*. 2024, 20^3^	sp. nov.	7C=BC543 (=DSM 46792=KCTC 59190)	27	[6]
*Comamonas squillarum* Shi *et al*. 2025, 7^3^	sp. nov.	PR12 (=JCM 35896=MCCC 1K08379)	37	[7]
*Crystallibacter* Plotnikova *et al*. 2025, 10^3^	gen. nov.	*Crystallibacter crystallopoietes* corrig.	13	[8]
*Crystallibacter crystallopoietes* corrig. (Ensign and Rittenberg 1963) Plotnikova *et al*. 2025, 10^3,8^	comb. nov. [basonym: *Arthrobacter crystallopoietes* Ensign and Rittenberg 1963 (Approved Lists 1980)]	VKM Ac-1107 (=DSM 20117=JCM 2522)^9^	13	[8]
*Crystallibacter degradans* Plotnikova *et al*. 2025, 12^3^	sp. nov.	SF27 (=LMG 33112=VKM Ac-2063)	13	[8]
*Crystallibacter permensis* Plotnikova *et al*. 2025, 11^3^	sp. nov.	B905 (=LMG 33113=VKM Ac-2550)	13	[8]
*Cupriavidus ulmosensis* Dawson *et al*. 2025, 18^3^	sp. nov.	CV2 (=CECT 30956=NCIMB 15506)	35	[9]
*Dechloromonas aquae* Wei *et al*. 2025, 7^3^	sp. nov.	Zy10 (=MCCC 1K08699=KCTC 72749)	32	[10]
*Enterococcus hailinensis* Wang *et al*. 2025, 7^3^	sp. nov.	N48-2 (=CCTCC AB 2024127=JCM 37000=LMG 33662)	28	[11]
*Flavobacterium ginsengiterrae* Kim *et al*. 2011, 345^10^	sp. nov.	DCY55 (=JCM 17337=KCTC 23319)	10	[12]
*Flavobacterium undicola* Chen *et al*. 2021, 997	sp. nov.	BBQ-18 (=BCRC 81050=KCTC 52810=LMG 30052)	11	[13]
*Gymnodinialimonas hymeniacidonis* Shin *et al*. 2024, 560	sp. nov.	202GB13-11 (=JCM 37088=KACC 23695)	34	[14]
*Gymnodinialimonas mytili* Shin *et al*. 2024, 561	sp. nov.	2305 UL16-5 (=JCM 37091=KACC 23693)	34	[14]
*Gymnodinialimonas ulvae* Shin *et al*. 2024, 561	sp. nov.	2307 UL20-7 (=KACC 23694=MCCC 1K09527)	34	[14]
*Halanaerocella* Gales *et al*. 2011, 570	gen. nov.	*Halanaerocella petrolearia*	9	[15]
*Halanaerocella petrolearia* Gales *et al*. 2011, 570	sp. nov.	S200 (=DSM 22693=JCM 16358)	9	[15]
*Halocatena halophila* (Tang *et al*. 2024) Zhu *et al*. 2025, 13^3^	comb. nov. [basonym: *Actinarchaeum halophilum* corrig. Tang *et al*. 2024]	YIM 93972 (=CGMCC 1.17467=DSM 46868)	24	[16]
*Isoptericola peretonis* Vidal-Verdú *et al*. 2025, 11^3^	sp. nov.	4D3 (=CECT 30740=DSM 115117)	19	[17]
*Jatrophihabitans lederbergiae* Nouioui *et al*. 2024, 21^3^	sp. nov.	AA-499 (=DSM 44399=KCTC 59193)^11^	27	[6]
*Kineococcus halophytocola* Thanompreechachai *et al*. 2025, 7^3^	sp. nov.	LSe6-4 (=NBRC 116401=TBRC 17798)	2	[18]
*Komagataeibacter piraceti* Karničnik *et al*. 2025, 14^3,12^	sp. nov.	Hr1 (=LMG 33628=ZIM B1167)	15	[19]
*Leptotrichia alba* Wang *et al*. 2025, 8^3^	sp. nov.	HSP-536 (=CGMCC 1.18096=JCM 36662=MCCC 1K09339)	42	[20]
*Leptotrichia mesophila* Wang *et al*. 2025, 8^3^	sp. nov.	HSP-342 (=CGMCC 1.18052=JCM 36567=MCCC 1K09338)	42	[20]
*Leptotrichia rugosa* Wang *et al*. 2025, 7^3^	sp. nov.	HSP-334 (=CGMCC 1.18095=JCM 36566=MCCC 1K09354)	42	[20]
*Leuconostoc aquikimchii* Kim *et al*. 2024, 1095	sp. nov.	MS01 (=JCM 37028=KACC 23748)	25	[21]
*Limnobacter olei* Liu *et al*. 2025, 8^3^	sp. nov.	P1 (=CCTCC AB 2019403=KCTC 72814)	12	[22]
*Microbacterium bandirmense* Saticioglu *et al*. 2025, 5^3^	sp. nov.	Mu-80 (=DSM 117210=LMG 33295)	29	[23]
*Microbacterium istanbulense* Saticioglu *et al*. 2025, 5^3^	sp. nov.	Mu-43 (=DSM 117065=LMG 33297)	29	[23]
*Microbacterium marmarense* Saticioglu *et al*. 2025, 5^3^	sp. nov.	Mu-86 (=DSM 117066=LMG 33293)	29	[23]
*Natronomonadaceae* Zhu *et al*. 2025, 12^3^	fam. nov.	*Natronomonas*	24	[16]
*Nocardia nepalensis* Aryal *et al*. 2025, 7^3^	sp. nov.	Aca13097 (=DSM 118930=NCCB 101050)	44	[24]
*Nocardiopsis lambiniae* Nouioui *et al*. 2024, 20^3^	sp. nov.	DCDM15A35 (=DSM 44743=KCTC 59192)	27	[6]
*Novacetimonas labruscae* Karničnik *et al*. 2025, 15^3,13^	sp. nov.	Jurk4 (=LMG 33630=ZIM B1166)	15	[19]
*Oceanimonas pelagia* Yang *et al*. 2024, 10^3,14^	sp. nov.	NTOU-MSR1 (=BCRC 81403=JCM 36023)	47	[25]
*Oceanisphaera submarina* Romanenko *et al*. 2025, 10^3^	sp. nov.	KMM 10153 (=KCTC 8836)	45	[26]
*Ornithinibacillus xuwenensis* Li *et al*. 2025, 8^3^	sp. nov.	16A2E (=GDMCC 1.4379=JCM 36753)	18	[27]
*Paenibacillus lacisoli* Xu *et al*. 2024, 8^3^	sp. nov.	JX-17 (=GDMCC 1.3962=KCTC 43568)	39	[28]
*Paenibacillus mesotrionivorans* Song *et al*. 2025, 6^3^	sp. nov.	P15 (=KCTC 43705=MCCC 1K09191)	38	[29]
*Polaribacter uvawellassae* Wanasinghe *et al*. 2025, 5^3^	sp. nov.	SLMDC-22 (=KCTC 102289=MCCC 1K09222)	30	[30]
*Providencia xihuensis* Dong *et al*. 2025, 11^3^	sp. nov.	SKLX074055 (=CCTCC AB2024038=NBRC 116649)	41	[31]
*Providencia zhejiangensis* Dong *et al*. 2025, 11^3^	sp. nov.	SKLX146130 (=CCTCC AB2024037=NBRC 116650)	41	[31]
*Pseudodesulfovibrio karagichevae* Bidzhieva *et al*. 2024, 14^3^	sp. nov.	9FUS (=KCTC 25498=VKM B-3654)	5	[32]
*Pseudodesulfovibrio methanolicus* Bidzhieva *et al*. 2024, 18^3^	sp. nov.	5S69 (=KCTC 25499=VKM B-3653)^15^	6	[33]
*Pseudomonas monachiensis* Yu *et al*. 2025, 15^3,16^	sp. nov.	PLb12A (=CCOS 2100=LMG 33494)^17^	22	[34]
*Pseudomonas rossensis* Snopková *et al*. 2025, 11^3,18^	sp. nov.	P2663 (=CCM 8880=LMG 32503)	31	[35]
*Pseudonocardia charpentierae* Nouioui *et al*. 2024, 20^3^	sp. nov.	DSM 45835 (=KCTC 59187)^19^	27	[6]
*Rarispira* Podosokorskaya *et al*. 2025, 7^3^	gen. nov.	*Rarispira pelagica*	40	[36]
*Rarispira pelagica* Podosokorskaya *et al*. 2025, 7^3^	sp. nov.	38H-sp (=DSM 100344=VKM B-2965)	40	[36]
*Robertmurraya mangrovi* Li *et al*. 2025, 9^3^	sp. nov.	31A1R (=GDMCC 1.4378=JCM 36937)	17	[37]
*Sabulicella glaciei* Liu and Xin 2024, 7^3^	sp. nov.	MDT2-1-1 (CGMCC 1.11170=NBRC 110485)	1	[38]
*Salinibaculum marinum* Zhu *et al*. 2025, 10^3^	sp. nov.	SYNS191 (=CGMCC 1.62607=JCM 36494)	24	[16]
*Salinibaculum rarum* Zhu *et al*. 2025, 10^3^	sp. nov.	KK48 (=CGMCC 1.19060=JCM 35607)	24	[16]
*Salinibaculum salinum* Zhu *et al*. 2025, 10^3^	sp. nov.	YCN56 (=CGMCC 1.62603=JCM 36493)	24	[16]
*Salinirubellus litoreus* Zhu *et al*. 2025, 11^3^	sp. nov.	SYNS196 (=CGMCC 1.62608=JCM 36495)	24	[16]
*Schlesneria sphaerica* Kulichevskaya *et al*. 2025, 7^3^	sp. nov.	T3-172 (=KCTC 102306=VKM B-3856)	21	[39]
*Sediminicurvatus* León *et al*. 2024, 16^3^	gen. nov.	*Sediminicurvatus halobius*	16	[40]
*Sediminicurvatus halobius* (Gong *et al*. 2023) León *et al*. 2024, 16^3^	comb. nov. [basonym: *Spiribacter halobius* Gong *et al*. 2023]	E85 (=KCTC 52699=MCCC 1H00230)	16	[40]
*Sphingosinicella rhizophila* Guo *et al*. 2025, 6^3^	sp. nov.	GR2756 (=CCTCC AB 2023102=CGMCC 1.61580=NBRC 116723)	23	[41]
*Spiribacter insolitus* León *et al*. 2024, 15^3^	sp. nov.	ag22IC4-189 (=CCM 9398=CECT 31037)	16	[40]
*Spiribacter pallidus* León *et al*. 2024, 15^3^	sp. nov.	SP10B (=CECT 9383=LMG 30156)^20^	16	[40]
*Streptococcus taoyuanensis* Lee *et al*. 2024, 4^3^	sp. nov.	ST2 (=BCRC 81374=NBRC 115928)	3	[42]
*Streptococcus vulneris* Chung *et al*. 2022, 6^3^	sp. nov.	DM3B3 (=BCRC 81288=NBRC 114638)	4	[43]
*Streptomonospora wellingtoniae* Nouioui *et al*. 2024, 21^3^	sp. nov.	CNQ 327 (=DSM 45055=KCTC 59191)	27	[6]
*Streptomyces althioticus* subsp. *althioticus* (Yamaguchi *et al*. 1957) Nouioui *et al*. 2024, 19^3,4^	comb. nov. [basonym: *Streptomyces althioticus* Yamaguchi *et al*. 1957 (Approved Lists 1980)]	ATCC 19724 (=BCRC 13686=CBS 463.68=CCRC 13686=CGMCC 4.1608=DSM 40092=IFO 12740=IFO 15956=JCM 4344=KCTC 9752=NBRC 12740=NBRC 15956=NRRL B-3981=NRRL ISP-5092=VKM Ac-705)	27	[6]
*Streptomyces althioticus* subsp. *attaecolombicae* Nouioui *et al*. 2024, 19^3^	subsp. nov.	Av26−2=DI-188 (=DSM 41972=KCTC 59178)	27	[6]
*Streptomyces antimycoticus* subsp. *antimycoticus* (Waksman 1957) Nouioui *et al*. 2024, 17^3,4^	comb. nov. [basonym: *Streptomyces antimycoticus* Waksman 1957 (Approved Lists 1980)]	ATCC 23880 (=CBS 660.68=CGMCC 4.1591=DSM 40284=IFO 12839=JCM 4228=JCM 4621=KCTC 9694=NBRC 12839=NRRL 2421=NRRL ISP-5284=VKM Ac-1824)	27	[6]
*Streptomyces antimycoticus* subsp. *sporoclivatus* (Preobrazhenskaya 1986 *ex* Krassilnikov 1970) Nouioui *et al.* 2024, 17^3^	comb. nov. [basonym: *Streptomyces sporoclivatus* (*ex* Krassilnikov 1970) Preobrazhenskaya 1986]	ATCC 43693 (=DSM 41461=JCM 9094=VKM Ac-315)^21^	27	[6]
*Streptomyces asiaticus* subsp. *asiaticus* (Sembiring *et al*. 2001) Nouioui *et al*. 2024, 16^3,4^	comb. nov. [basonym: *Streptomyces asiaticus* Sembiring *et al*. 2001]	A10598 (=ATCC 15166=DSM 41524)	27	[6]
*Streptomyces asiaticus* subsp. *ignotus* Nouioui *et al*. 2024, 16^3^	subsp. nov.	A10598 (=ATCC 15166=DSM 41524)	27	[6]
*Streptomyces boetiae* Nouioui *et al*. 2024, 19^3^	sp. nov.	SCRIPP CNQ 259 (=DSM 44917=KCTC 59173)	27	[6]
*Streptomyces bugiae* Nouioui *et al*. 2024, 16^3^	sp. nov.	SF-1084 (=DSM 41528=ATCC 21722)^22^	27	[6]
*Streptomyces chisholmiae* Nouioui *et al*. 2024, 19^3^	sp. nov.	SCRIPP CNJ 962 (=DSM 44915=KCTC 59194)	27	[6]
*Streptomyces doebereineriae* Nouioui *et al*. 2024, 17^3^	sp. nov.	13-A30 (=DSM 41640=KCTC 59177)	27	[6]
*Streptomyces doudnae* Nouioui *et al*. 2024, 18^3^	sp. nov.	Sol5a-2 (=DSM 41981=KCTC 59175)	27	[6]
*Streptomyces dubilierae* Nouioui *et al*. 2024, 18^3^	sp. nov.	S6 (=DSM 41921=KCTC 59180)	27	[6]
*Streptomyces edwardsiae* Nouioui *et al*. 2024, 17^3^	sp. nov.	31-A2 (=DSM 41636=KCTC 59179)	27	[6]
*Streptomyces evansiae* Nouioui *et al*. 2024, 18^3^	sp. nov.	SA3-ActF (=DSM 41979=KCTC 59185)	27	[6]
*Streptomyces gibsoniae* Nouioui *et al*. 2024, 17^3^	sp. nov.	ATB-26 (=DSM 41699=KCTC 59183)	27	[6]
*Streptomyces gottesmaniae* Nouioui *et al*. 2024, 14^3^	sp. nov.	Tü 2253 (=DSM 3412=KCTC 59181)	27	[6]
*Streptomyces hazeniae* Nouioui *et al*. 2024, 18^3^	sp. nov.	SCSIO 10374 (=DSM 42041=KCTC 59186)	27	[6]
*Streptomyces hintoniae* Nouioui *et al*. 2024, 16^3^	sp. nov.	DSM 41014 (=KCTC 59176)^23^	27	[6]
*Streptomyces johnsoniae* Nouioui *et al*. 2024, 18^3^	sp. nov.	CNB 984 (=DSM 41886=KCTC 59171)	27	[6]
*Streptomyces kroppenstedtii* Nouioui *et al*. 2024, 14^3^	sp. nov.	DSM 40484 (=KCTC 59371)	26	[44]
*Streptomyces kutzneri* Nouioui *et al*. 2024, 14^3^	sp. nov.	DSM 40907 (=KCTC 59373)	26	[44]
*Streptomyces lancefieldiae* Nouioui *et al*. 2024, 15^3^	sp. nov.	Tü 41 (=DSM 40712=NRRL 2835)	27	[6]
*Streptomyces litchfieldiae* Nouioui *et al*. 2024, 20^3^	sp. nov.	SCRIPP CNR954 (=DSM 44938=KCTC 59172)	27	[6]
*Streptomyces lonegramiae* Nouioui *et al*. 2024, 17^3^	sp. nov.	A-130 (=DSM 41529=ATCC 21840)^24^	27	[6]
*Streptomyces millisiae* Nouioui *et al*. 2024, 20^3^	sp. nov.	SCRIPP CNQ 703 (=DSM 44918=KCTC 59174)	27	[6]
*Streptomyces mooreae* Nouioui *et al*. 2024, 16^3^	sp. nov.	SF-1293 (=DSM 41527=ATCC 21705)	27	[6]
*Streptomyces salyersiae* Nouioui *et al*. 2024, 18^3^	sp. nov.	157/96 (=DSM 41770=KCTC 59182)	27	[6]
*Streptomyces stackebrandtii* Nouioui *et al*. 2024, 14^3^	sp. nov.	DSM 40976 (=Gütt 467=KCTC 59369)	26	[44]
*Streptomyces stephensoniae* Nouioui *et al*. 2024, 16^3^	sp. nov.	DSM 40932 (=ATCC 13741)^25^	27	[6]
*Streptomyces winkii* Nouioui *et al*. 2024, 15^3^	sp. nov.	Sandoz 59283 (=DSM 40971=KCTC 59372)	26	[44]
*Streptomyces zaehneri* Nouioui *et al*. 2024, 14^3^	sp. nov.	DSM 40713 (=ETH 21510=KCTC 59370=Tü 43)	26	[44]
*Tenuifilum osseticum* Podosokorskaya *et al*. 2025, 6^3,26^	sp. nov.	4138-str (=KCTC 25386=UQM 41477=VKM B-3628)	7	[45]
*Tepidanaerobacter acetatoxydans* Westerholm *et al*. 2011, 265^25^	sp. nov.	Re1 (=DSM 21804=JCM 16047)	8	[46]
*Vibrio chaetopteri* Kurilenko *et al*. 2025, 14^3^	sp. nov.	CB1-14 (=KCTC 92790=KMM 8419)	46	[47]
*Winmispira* Podosokorskaya *et al*. 2025, 7^3^	gen. nov.	*Winmispira thermophila*	40	[36]
*Winmispira thermophila* (Aksenova *et al*. 1992) Podosokorskaya *et al*. 2025, 7^3^	comb. nov. [basonym: *Spirochaeta thermophila* Aksenova *et al*. 1992]	Z-1203 (=ATCC 700085=DSM 6578	40	[36]
*Winmispiraceae* Podosokorskaya *et al*. 2025, 7^3^	fam. nov.	*Winmispira*	40	[36]
*Winmispirales* Podosokorskaya *et al*. 2025, 7^3^	ord. nov.	*Winmispira*	40	[36]

For references to Validation Lists 1-71, see *Int J Syst Bacteriol* **49** (1999) 1325. Lists 72-197 were published in *Int J Syst Evol Microbiol* **50** (2000) 3, 423, 949, 1415, 1699, 1953; and **51** (2001) 1, 263, 793, 1229, 1619, 1945; and **52** (2002) 3, 685, 1075, 1437, 1915; and **53** (2003) 1, 373, 627, 935, 1219, 1701; and **54** (2004) 1, 307, 631, 1005, 1425, 1909; and **55** (2005) 1, 547, 983, 1395, 1743, 2235; and **56** (2006) 1, 499, 925, 1459, 2025, 2507; and **57** (2007) 1, 433, 893, 1371, 1933, 2449; and **58** (2008) 1, 529, 1057, 1511, 1993, 2471; and **59** (2009) 1, 451, 923, 1555, 2129, 2647; and **60** (2010) 1, 469, 1009, 1477, 1985, 2509; **61** (2011) 1, 475,1011, 1499, 2025, 2563; and **62** (2012) 1, 473, 1017, 1443, 2045, 2549; and **63** (2013) 1, 797, 1577, 2365, 3131, 3931; and **64** (2014) 1, 693, 1455, 2184, 3603; and **65** (2015), 1, 741, 1112, 2017, 2777, 3767; and **66** (2016) 1, 1603, 1913, 2463, 3761, 4299; and **67** (2017) 1, 529, 1095, 2075, 3140, 4291; and **68** (2018), 1, 693, 1411, 2130, 2707, 3379; and **69** (2019), 5, 597, 1247, 1844, 2627, 3313, and **70** (2020) 1, 1443, 2960, 4043, 4844, 5596. Lists 197 223 were published in *Int J Syst Evol Microbiol* **71** (2021) 004600, 004688, 004773, 004846, 004943, 005096; and **72** (2022) 005167, 005260, 005331, 005422, 005517, 005592; and **73** (2023) 005709, 005812, 005845, 005931, 005997, 006080; and **74** (2024) 006173, 006229, 006275, 006398, 006452, 006501; and **75** (2025) 006562, 006640, 006699.

1Abbreviations of culture collections cited in this list can be found at https://lpsn.dsmz.de/text/collections

2Priority number assigned according to the date of the documentation and request for validation are received.

3The journal in which the name was effectivey published does not have continuous pages.

4Automatically created as required by Rule 40d of the ICNP.

5The preferred etymology is N.L. masc. adj. *groningensis*, …

6The nomenclature reviewers have corrected the epithet *kampingiae* to *kampingae* (kam.pin’gae. N.L. gen. n. *kampingae*, named in honour of … Greetje Kampinga …).

7The preferred etymology is N.L. masc. adj. *martiniensis*, …

8 The list editors corrected *crystallifaciens* to *crystallopoietes* to comply with Rule 41a of the ICNP.

9The effective publication states that the type strain was also deposited as ATCC 1548, CIP 102717, LMG 3819 and NBRC 14235, but no documentation was supplied.

10The preferred etymology is N.L. neut. n. *ginsengum*, ginseng; L. fem. n. *terra*, soil; N.L. gen. n. *ginsengiterrae*,

11The effective publication states that the type strain was also deposited as IFAM AA-499, but no documentation was supplied.

12The preferred syllabification and etymology are pir.a.ce’ti. L. neut. n. *pirum*, pear; L. neut. n. *acetum*, vinegar; N.L. gen. n. *piraceti*, of pear vinegar,

13The preferred etymology is L. fem. n. *labrusca*, wild vine or grape; L. gen. n. *labruscae*, from *Vitis labruscae*, indicating its origin from vinegar made with apples and grapes of the *Vitis labruscae* variety.

14The protologue heading must state sp. nov. instead of sp. Nov., and the etymology must state L. fem. adj. instead of L. f. adj.

15The effective publication states that the type strain was also deposited as UQM 41509, but no documentation was supplied.

16The etymology must state N.L. fem. adj. instead of N.L. gen. n.

17The effective publication states that the type strain was also deposited as DSM 116615, but no documentation was supplied.

18The etymology was given at the end of the protologue, so that the order required by Rule 27 was not implemented.

19The effective publication states that the type strain was also deposited as CPCC 203558, but no documentation was supplied.

20The effective publication states that the type strain was also deposited as IBRC-M 11160, but no documentation was supplied.

21The effective publication states that the type strain was also deposited as INMI 97 and NBRC 100767, but no documentation was supplied.

22The effective publication states that the type strain was also deposited as FERM-P 602, but no documentation was supplied.

23The effective publication states that the type strain was also deposited as IMRU 3066, but no documentation was supplied.

24The effective publication states that the type strain was also deposited as FERM-P 639, but no documentation was supplied.

24The effective publication states that the type strain was also deposited as ATCC 13793, CBS 372.58, ETH 24437 and NRRL B-1354, but no documentation was supplied.

25The etymology must state N.L. fem. adj. instead of N.L. neut. Adj.

26The protologue heading must be *Tepidanaerobacter acetatoxydans* instead of *T. acetatoxydans*.

## Correction to Validation List no. 220 [48]

In Validation List no. 220, the type strain designations for *Mycobacterium barrassiae* were given as ‘N7 (=CCUG 50398=NBRC 113646)’. Correct is ‘N7 (=CCUG 50398=CIP 108545)’.

We thank S. Turner (NCBI, NIH, Bethesda, MD, USA) for alerting us to the error in Validation List no. 220.

## Abbreviations

ICNP: International Code of Nomenclature of Prokaryotes
IJSEM: International Journal of Systematic and Evolutionary Microbiology
